# Kernel-based whole-genome prediction of complex traits: a review

**DOI:** 10.3389/fgene.2014.00363

**Published:** 2014-10-16

**Authors:** Gota Morota, Daniel Gianola

**Affiliations:** ^1^Department of Animal Science, University of Nebraska-LincolnLincoln, NE, USA; ^2^Department of Animal Sciences, University of Wisconsin-MadisonMadison, WI, USA; ^3^Department of Biostatistics and Medical Informatics, University of Wisconsin-MadisonMadison, WI, USA; ^4^Department of Dairy Science, University of Wisconsin-MadisonMadison, WI, USA

**Keywords:** whole-genome prediction, kernel methods, semi-parametric regression, spatial distance, SNP

## Abstract

Prediction of genetic values has been a focus of applied quantitative genetics since the beginning of the 20th century, with renewed interest following the advent of the era of whole genome-enabled prediction. Opportunities offered by the emergence of high-dimensional genomic data fueled by post-Sanger sequencing technologies, especially molecular markers, have driven researchers to extend Ronald Fisher and Sewall Wright's models to confront new challenges. In particular, kernel methods are gaining consideration as a regression method of choice for genome-enabled prediction. Complex traits are presumably influenced by many genomic regions working in concert with others (clearly so when considering pathways), thus generating interactions. Motivated by this view, a growing number of statistical approaches based on kernels attempt to capture non-additive effects, either parametrically or non-parametrically. This review centers on whole-genome regression using kernel methods applied to a wide range of quantitative traits of agricultural importance in animals and plants. We discuss various kernel-based approaches tailored to capturing total genetic variation, with the aim of arriving at an enhanced predictive performance in the light of available genome annotation information. Connections between prediction machines born in animal breeding, statistics, and machine learning are revisited, and their empirical prediction performance is discussed. Overall, while some encouraging results have been obtained with non-parametric kernels, recovering non-additive genetic variation in a validation dataset remains a challenge in quantitative genetics.

## 1. Introduction

Six years after the rediscovery of Mendel's laws of heredity, Toyama Kametaro's experimental work on silkworm breeding showed the first case of Mendelian inheritance in animals (Onaga, 2010). Yule (1902) made a first attempt at expanding Mendelian theory to factor in quantitative variation, followed by a seminal paper by Fisher (1918) nearly a century ago (Plutynski, 2006). A fundamental concept in quantitative genetics is that of linking genotypes and phenotypes through genetic similarity among individuals, i.e., covariance between relatives (Wright, 1921). The main focus today is to statistically model variation in DNA sequences influencing phenotypic variation in quantitative traits, rather than understanding the biological pathways that are associated with selective genes of interest, which falls in the domain of molecular genetics. The discipline of genome-based prediction is a subfield of quantitative genetics that aims to predict unobserved values by regressing phenotypes on measures of genetic resemblance, obtained from DNA data. Although early attempts took place in the 80's (e.g., Fernando and Grossman, 1989; Lande and Thompson, 1990), implementation of genome-based prediction was largely hindered by scarce molecular information. It was just recently that the subject began to attract widespread attention following the availability of rich DNA variation data spanning the whole genome (e.g., Meuwissen et al., 2001; Gianola et al., 2003). This approach continues to progress rapidly and has been fruitfully applied to a variety of quantitative traits of agronomic importance in animals (e.g., Hayes et al., 2009; VanRaden et al., 2009) and plants (e.g., Crossa et al., 2014). The objective of “genome-enabled selection” is to predict responses by capturing additive genetic effects that may have implication in choosing individuals as parents of the next generation. Statistical methodologies tailored to this application have been reviewed in a number of papers (e.g., Gianola et al., 2009; Calus, 2010; de los Campos et al., 2013a; Gianola, 2013; Meuwissen et al., 2013).

Concurrently, whole-genome prediction of “total” genetic effects has been motivated by the fact that phenotypes and genotypes may not be linearly related and that the additivity assumption, even though useful, is violated (Gianola et al., 2010). Importance of predicting non-additive genetic effects comes into the picture in exploitation of heterosis, mate allocation, cross-breeding, and precision mating in breeding contexts, and more crucially when prediction of phenotypes is a primal point, such as disease outcome. The objective of this article is to provide a survey of emerging statistical approaches based on kernel methods with emphasis on “prediction” rather than on genome-enabled selection for breeding. Special focus is placed on a semiparametric kernel methodology that condenses genealogical or genomic information into a positive (semi) definite relationship matrix. We highlight insights collected from research conducted in recent years and suggest potential future directions in this area.

In the next section, we go through statistical models involving the use of kernels. Subsequently, we review a variety of kernel matrices that have been applied to date. We then survey applications of kernel methods to real data in a whole-genome prediction framework, and concluding remarks are given in the final section.

## 2. Kernel-based regression methods

We first review kernel-based prediction models being used for prediction using genomic data. Our aim is to approximate an unknown “true” genetic signal **g** with a certain function of a marker genotypes matrix *f*(**X**) that maps these genotypes to responses (**y**). The data generating model is then **y** = *f*(**X**) + **ϵ**, where **y** is the vector of phenotypes and **ϵ** is a vector of residuals. In general, all kernel methods differ from each other in the choice of the mapping function *f*(.) and the type of regularization used to balance goodness of fit and complexity, as discussed later.

### 2.1. Genomic BLUP

Our main interest is to identify the model that gives best prediction among a set of candidate models. To find the best predictive function *f*(**X**) there are a few things that we need to set up prior to the search. One is whether we should impose a restriction on the search space or not. The parameter space is a space where all models are characterized by parameters. In a linear model, where response values have linear relationship with respect to the parameters, though, it could be non-linear on covariates such as in the case of polynomial regression. Once the parameters are given, all models are distinct. For example, the two linear models would be

$$\begin{array}{l} \text{Model 1: }y=a_{1}+b_{1}x_{1}+c_{1}x_{2}\\ \text{Model 2: }y=a_{2}+b_{2}x_{1}+c_{2}x_{2}. \end{array}$$

If we give values to parameters *a*_*i*_, *b*_*i*_, and *c*_*i*_ then Models 1 and 2 can be differentiable. In other words, we just need to fill out the unknown parameters of the given models. The best linear unbiased predictor (BLUP) is a procedure for filling the unknown values (Henderson, 1975).

Suppose underlying signal is given by **y** = **g** + **ϵ**. Here, **g** is the true unknown genetic signal with **g** ~ *N*(0, **Γ**σ^2^_**g**_), where **Γ** is a “true” genomic relationship matrix among animals, i.e., at the quantitative trait loci affecting the trait. Since the latter are unknown, we approximate the vector of genetic values **g** with a linear function such that **y** = **Xβ** + **ϵ**, where **X** is an *n* (number of animals) × *m* (number of markers) matrix of additive marker genotypes potentially centered or scaled; **β** is a vector of regression coefficients on marker genotypes; and *ϵ* is a residual that includes model misspecification and environmental effects not considered in this analysis. Under the independence assumption between **g** and **ϵ**, the variance-covariance matrix of **y** is

$$\begin{array}{l} V_{y}=V_{g}+V_{\epsilon }\\ \;=XX^{T}\sigma_{\beta }^{2}+I\sigma_{\epsilon }^{2} \end{array}$$

often assuming that **β** ~ *N*(0, **I**σ^2^_**β**_) and **ϵ** ~ *N*(0, **I**σ^2^_**ϵ**_). Here, **V**_*g*_ = **XX**^*T*^σ^2^_β_ is the covariance matrix “due to” markers. The problem is to predict **g** such that two conditions are met: (1) E(ĝ) = E(**g**) = 0, and (2) var(*ĝ*_*i*_ − *g*_*i*_) is minimum for *i* over all linear functions that satisfy the unbiasedness condition (1). If normality is assumed, the BLUP of **g**(**ĝ**) is the conditional mean of **g** given the data, and takes the form

(1) $$\begin{array}{l} BLUP(\hat{g})=E(g|y)=E[g]+Cov\left(g,y^{T}\right)Var{\left(y\right)}^{-1}[y-E(y)]\\ \;=Cov\left(X\beta ,y^{T}\right)\cdot V_{y}^{-1}y\\ \;=XX^{T}\sigma_{\beta }^{2}{\left[XX^{T}\sigma_{\beta }^{2}+I\sigma_{\epsilon }^{2}\right]}^{-1}y\\ \;={\left[I+{\left(XX^{T}\right)}^{-1}\frac{\sigma_{\epsilon }^{2}}{\sigma_{\beta }^{2}}\right]}^{-1}y, \end{array}$$

assuming that **XX**^*T*^ is invertible. Here, *Cov*(**X**) = **XX**^*T*^ is a covariance matrix of marker genotypes (provided that *X* is centered), often considered to be the simplest form of additive genomic relationship kernel, **G**. We can refine this kernel by relating genetic variance σ^2^_*g*_ and marker variance σ^2^_β_ under the following assumptions. Again, assume genetic value is parameterized as *g*_*i*_ = ∑*x*_*ij*_β_*j*_, where both *x* and β are treated as random and independent. Under Hardy-Weinberg equilibrium, *E*(*x*_*ij*_) = 2*p*_*j*_ and *Var*(*x*_*ij*_) = 2*p*_*j*_(1 − *p*_*j*_), where *p*_*j*_ is the minor allele frequency of locus *j*, and assuming linkage equilibrium of markers (all loci are mutually independent),

$$\sigma_{g}^{2}\;=\sum_{j}2p_{j}(1-p_{j})\cdot \sigma_{\beta_{j}}^{2}.$$

Under the homogeneous marker variance assumption, one obtains the relationship

(2) $$\sigma_{\beta }^{2}\;=\frac{\sigma_{g}^{2}}{2\sum_{j}p_{j}(1-p_{j})}.$$

Replacing σ^2^_β_ in Equation (1) with (2), we get

(3) $$\begin{array}{l} BLUP(\hat{g})={\left[I+{\left(XX^{T}\right)}^{-1}\frac{\sigma_{\epsilon }^{2}}{\frac{\sigma_{g}^{2}}{2\sum_{j}p_{j}(1-p_{j})}}\right]}^{-1}y\\ \;={\left[I+G^{-1}\frac{\sigma_{\epsilon }^{2}}{\sigma_{g}^{2}}\right]}^{-1}y \end{array}$$

where $G=\frac{XX^{T}}{2\sum_{j}p_{j}(1-p_{j})}$ is known as the first **G** matrix introduced in VanRaden (2008).

Likewise, σ^2^_*g*_ = *m*σ^2^_β_ if it is assumed that all markers have variance 1 (following standardizing marker genotypes) so the marked genetic variance is given by the sum of individual marker variances. With this, σ^2^_β_ = σ^2^_*g*_/*m*, and **G** in Equation (3) becomes the second additive genomic relationship kernel of VanRaden (2008), $\frac{XX^{T}}{m}$. One can also approximate **Γ** with a pedigree-based relationship kernel **A** instead of **G**, embedding correlations due to expected additive genetic inheritance. How close **G** approximates **Γ** depends on observed or tagged causal loci in the data. A third type of kernel matrix is a linear combination λ**A** + (1 − λ)**G**, were 0 < λ < 1 is the weight placed on **A** relative to **G** (e.g., Rodríguez-Ramilo et al., 2014).

### 2.2. Reproducing kernel Hilbert spaces regression

Genomic BLUP (GBLUP) is a linear model characterized by parameters that relate to additive quantitative genetics theory. We now extend the search space by eliminating some restrictions. For instance, semiparametric regressions identify functional forms “before as well as in the midst” of the fitting process (Berk, 2008). A reproducing kernel Hilbert spaces (RKHS) regression represents this type of approach. Here, the true genetic signal **g** = {*g*_*i*_} is approximated as an unknown function of genetic effects *g*(**x**_*i*_), contrary to assuming **g** = **Xβ** as in the case of GBLUP. This *g*(**x**_*i*_) function is viewed as the conditional expectation *E*(**y**_*i*_|**x** = **x**_*i*_), that is, the average phenotypic value of individuals possessing marker genotype **x**_*i*_ without restricting the form of *g*_*i*_. RKHS regression proceeds by searching a function and uses the residual sum of squares as a loss function, and assigns the squared norm of **g** under a Hilbert space as a penalty. The objective function to be minimized with respect to **g** is

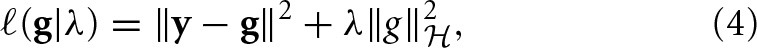



where λ is a regularization parameter and 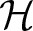 represents a Hilbert space, very rich class of functions. While there are countless candidates for **g** in non-parametric regression, with this setting, the representer theorem developed by Kimeldorf and Wahba (1971) guarantees that the optimizer will be in the span of the functions indexed by the observed covariates. This implies that the objective function reduces to a linear function **Kα**, where **K** is an *n* × *n* kernel constructed from the observed data and **α** is an *n* × 1 vector of regression coefficients to be inferred, e.g., by minimizing

(5) $$\ell \left(\alpha |\lambda \right)=\left(y-K\alpha \right)\prime \left(y-K\alpha \right)+\lambda \alpha^{\prime }K\alpha .$$

Equation (5) is minimized by taking its derivative with respect to **α** and setting to 0 to obtain:

(6) $$\hat{\alpha }={\left(K+\lambda I\right)}^{-1}y,$$

so that the predicted genetic value is given by **ĝ** = **K**$\hat{\alpha }$; this requires λ to be known.

This regression procedure is also known as kernel ridge regression in machine learning, and was first introduced in quantitative genetics by Gianola et al. (2006) and Gianola and van Kaam (2008) in the context of a mixed effects model with a Bayesian treatment. Efficient Gibbs sampling algorithms for RKHS regression have been developed by de los Campos et al. (2010) by exploiting the eigendecomposition of kernels. When the first term in Equation (4) is replaced by the “epsilon-insensitive” loss function, this is equivalent to support vector regression (Moser et al., 2009; Long et al., 2011). Hence, RKHS represents a general and powerful paradigm.

Note that the representer theorem requires assigning the *L*2 norm (Euclidean norm) to regularize the regressions **α**. This regularizer assures that optimal solutions lie in a finite-dimensional rather than in an infinite-dimensional space. In practical situations, sparsity induced by the *L*1 norm (Manhattan norm) may be preferable. This is equivalent to replacing **α′Kα** in Equation (5) with $\sum_{i=1}^{n}\left|\alpha_{i}\right|$. However, this violates assumptions of the representer theorem and it no longer guarantees that the optimizer is given by a linear combination of the data points. Nevertheless, it is conceivable that norms other than *L*2 may deliver better predictions.

### 2.3. RKHS and BLUP

The important connection between RKHS regression and BLUP was brought up first by Harville (1983); Robinson (1991) and de los Campos et al. (2009) and this part of the review follows their work closely. Suppose we approximate a genetic signal with a vector of additive effects and assume a single record per individual. The model is

(7) y =Xβ+Iα+ϵ

where **y** is the response variable, **X** is an incidence matrix linking the response to some nuisance effects; **β** and **α** are regression coefficients and **ϵ** is a residual that includes model misspecification and environmental effects not considered in this analysis. The two random components of the model follow the distribution **α** ~ *N*(0, **A**σ^2^_α_) and **ϵ** ~ *N*(0, **I**σ^2^_ϵ_), where σ^2^_α_ is the additive genetic variance and **A** is the numerator relationship matrix between individuals (relationships in the absence of inbreeding). Henderson's mixed model equations (MME) are

(8) $$\left[\begin{array}{cc} X^{T}X & X^{T}\\ X & I+A^{-1}\frac{\sigma_{e}^{2}}{\sigma_{a}^{2}} \end{array}\right]\left[\begin{array}{c} \hat{\beta }\\ \hat{\alpha } \end{array}\right]=\left[\begin{array}{c} X^{T}y\\ y \end{array}\right].$$

Now, transform additive genetic effects into α^*^ = **A**^−1^**α** (assuming that **A**^−1^ exists) so that Equation (7) is reexpressed as

$$y\;=X\beta +A\alpha^{*}+\epsilon ,$$

where α^*^ ~ *N*(0, **A**^−1^σ^2^_α_), and the corresponding MME are

(9) $$\left[\begin{array}{cc} X^{T}X & X^{T}A\\ AX & A^{2}+A\frac{\sigma_{e}^{2}}{\sigma_{a}^{2}} \end{array}\right]\left[\begin{array}{c} \hat{\beta }\\ {\hat{\alpha }}^{*} \end{array}\right]=\left[\begin{array}{c} X^{T}y\\ Ay \end{array}\right],$$

because **A** is symmetric. By multiplying the α^*^ equation by **A**^−1^, one obtains

(10) $$\left[\begin{array}{cc} X^{T}X & X^{T}A\\ X & A+I\frac{\sigma_{e}^{2}}{\sigma_{a}^{2}} \end{array}\right]\left[\begin{array}{c} \hat{\beta }\\ {\hat{\alpha }}^{*} \end{array}\right]=\left[\begin{array}{c} X^{T}y\\ y \end{array}\right].$$

If phenotypes are pre-corrected for systematic effects (thus **β** = 0) a priori, then Equation (10) reduces to

(11) $$\begin{array}{l} {}[A+\lambda I]\alpha^{*}=y\\ \;{\hat{\alpha }}^{*}={\left(A+\lambda I\right)}^{-1}y \end{array}$$

where $\lambda =\frac{\sigma_{e}^{2}}{\sigma_{a}^{2}}$ is a regularization parameter. Replacing the pedigree-based relationship kernel **A** with a more general term **K** yields α^*^ = [**K** + λ**I**]^−1^**y**. Since BLUP is linearly invariant, the BLUP of **α** is given by $\hat{\alpha }$ = **A**^−1^ $\hat{\alpha }$^*^ = **A**^−1^ (**A** + λ**I**)^−1^**y**. This is equivalent to Equation (6) and is the Bayesian kernel ridge regression employed in de los Campos et al. (2010) and Morota et al. (2013). Thus, BLUP of additive effects can be viewed as a regression on pedigree or on additive genomic relationship kernels. It is interesting to note that BLUP, developed in animal breeding, is a special case of RKHS, developed in statistics.

### 2.4. Connection between the kernel and the matrix of genotypes

GBLUP is also linked to BLUP of marker regression coefficients. Here, we show how BLUP of regression on markers and BLUP of additive genetic values are related to each other. This relationship was first shown by Henderson (1977) in the context of predicting BLUP of non-phenotyped animals, and was rediscovered recently (e.g., Goddard, 2009). Suppose that the phenotype-genotype mapping function is **y** = **g** + **ϵ**, where the genetic effect is parameterized as **g** = **Xβ**. Here, **X** is the matrix of marker genotypes and **β** is the marker allele substitution effects. Then, the conditional expectation of **β** given **y** assuming known dispersion parameters and **β** ~ *N*(0, **I**σ^2^_β_) is

$$\begin{array}{l} BLUP(\beta )=E(\beta |y)=Cov(\beta ,y)Var{\left(y\right)}^{-1}[y-X\beta ]\\ \;=Cov(\beta ,X\beta ){\left[XX^{T}\sigma_{\beta }^{2}+I\sigma_{\epsilon }^{2}\right]}^{-1}y\\ \;=\sigma_{\beta }^{2}X^{T}{\left[XX^{T}\sigma_{\beta }^{2}+I\sigma_{\epsilon }^{2}\right]}^{-1}y\\ \;=\sigma_{\beta }^{2}X^{T}{\left(XX^{T}\right)}^{-1}{\left[\sigma_{\beta }^{2}I+{\left(XX^{T}\right)}^{-1}\sigma_{\epsilon }^{2}\right]}^{-1}y\\ \;=X^{T}{\left(XX^{T}\right)}^{-1}{\left[I+{\left(XX^{T}\right)}^{-1}\frac{\sigma_{\epsilon }^{2}}{\sigma_{\beta }^{2}}\right]}^{-1}y. \end{array}$$

Using Equation (3), we get

$$\begin{array}{l} BLUP(\beta )=X^{T}{\left(XX^{T}\right)}^{-1}{\left[I+G^{-1}\frac{\sigma_{\epsilon }^{2}}{\sigma_{g}^{2}}\right]}^{-1}y\\ \;=X^{T}{\left(XX^{T}\right)}^{-1}BLUP(g). \end{array}$$

Thus, once we obtain **ĝ** from GBLUP, BLUP of marker coefficients is given by $\hat{\beta }$ = **X**^*T*^ (**XX**^*T*^)^−1^**ĝ**. We arrive at the same prediction regardless of whether we start from the genotype matrix **X** or from the **g**. Note that marker-based regressions can also be “kernelized” when the squared norm of **β** is assigned as penalty function. Ridge regression with markers treated as random effects is known to be equivalent to BLUP, i.e., RR-BLUP (Ruppert et al., 2003). However, the least absolute shrinkage and selection operator (LASSO) does not satisfy this condition. Although RR-BLUP and GBLUP are mathematically equivalent, predictive ability has differed when applied to real data. For example, Zhong et al. (2009) and Massman et al. (2013) observed a superiority of RR-BLUP over GBLUP in the presence of strong LD, and Habier et al. (2013) reported that GBLUP was not able to utilize short-range LD information for prediction.

### 2.5. RKHS family

It should be noted that the family of RKHS also extends to independently developed spatial statistics regression (Stein, 1999). Kriging is a variant of BLUP used for predicting values of variables distributed over a space (Stein, 1999). It is a spatial regression technique based on spatial associations at various locations. We first revisit the space which kriging takes place when applied to genomic data.

#### 2.5.1. Spatial genotypic structure

Suppose that all individuals in a sample have been genotyped for biallelic SNPs spanned over the whole genome, with these SNPs used for genome-enabled prediction of quantitative traits. The number of possible genotypic configurations with *m* SNPs is 3^*m*^, which is an enormous number when *m* exceeds the thousands. Characterizing the metric space governed by these SNP predictors prior to the analysis is of importance in a spatial prediction problem. Recently, Morota et al. (2013) used SNP codes as coordinates of genotypes in *m*-dimensional spaces. Therein, an *m*-dimensional grid graph with vertices representing a vector of individual's genotypes was proposed as a spatial structure of genotypes. This *m*-dimensional non-Euclidean metric space can be constructed once the total number of SNP genotypes is obtained. Then, each sampled individual in the data set is placed at one of the vertex on this space. With genotypes coded as (0, 1, 2) for “aa,” “Aa,” and “AA,” respectively, two vertices are adjacent if and only if codes at just one SNP locus differ by 1.

In standard regression analysis, independent and identically distributed (i.i.d.) observations is a common assumption while, in kriging, spatially correlated random variables are considered (Isaaks and Srivastava, 1990). The mixed models of Henderson (1975) do not take spatial association of individuals into account. However, in genetics it is known that individuals are often genetically related to each other, especially in animal breeding. For this reason, the numerator relationship matrix **A** or the genomic relationship matrix **G** among individuals are employed. With this, the set of additive effects in a sample of individuals is assumed to follow a multivariate normal distribution with a zero mean vector and a covariance matrix that is proportional to **A** or **G**, instead of the identity matrix **I**. On the other hand, kriging explicitly assumes at the onset that observed values are spatially correlated.

Most of observed data are not randomly sampled. This is particularly so in animal breeding, where individuals genotyped tend to be highly related due to intense artificial selection. When we embed these samples into a 3^*m*^ grid graph, it is unlikely that points will be uniformly distributed in this space. We would expect to see clusters of individuals because of their genotypic similarity due to selection, or no clusters at all in certain regions. A kriging system attempts to smooth the irregular spatial variation in the *p*-dimensional space, to arrive at a better prediction of outcomes (Isaaks and Srivastava, 1990). The main goal here is to predict phenotypes at unsampled vertices using data obtained at limited number of neighboring spatial locations. The kriging system, which performs an interpolation on this grid space, is briefly explained in the following subsection.

#### 2.5.2. Kriging

For simplicity, only ordinary kriging is reviewed. Suppose that available phenotypes are viewed as the result of some stochastic process with *y*_*i*_ = *g*(**x**) + *e*_*i*_ having the measurement on individual *i* (*i* = 1, ···, *n*) at point *x*_*i*_. A random field *g* is a random function on a space 𝔻, which in our case is the *p*-dimensional grid graph ℤ^*p*^_3_ of all SNP genotypes, and *x*_*i*_ and *e*_*i*_ are the *i*th observed vertex and residual. In other words, *y*_*i*_ is a response value associated with a spatial point location *x*_._ which is a vertex. We assume second order stationarity in the observations, that is, the mean is constant but unknown and the covariance depends only on the “distance” between any of two vertices (∥*x*_*i*_ − *x*_*j*_∥), but not on the locations *per se*. We wish to predict the phenotype of an individual having vertex *x*_0_ using all available data. Kriging is the best predictor (Henderson, 1973) in the mean-squared error sense,

$$\arg\;\underset{{\hat{y}}_{0}}{\min}\{\text{E}{\left[\underset{predicted}{\underset{︸}{{\hat{y}}_{0}}}-\underset{unobserved}{\underset{︸}{y_{0}}}\right]}^{2}\}$$

where *y*_0_ is the phenotype at vertex *x*_0_ to be predicted and *ŷ*_0_ is its predictor.

As in BLUP, in kriging, the predictor is restricted to the class of linear functions of the data and given by a weighted linear combination of all available observations, that is,

(12) $${\hat{y}}_{0}=\sum_{i\;=\;1}^{n}w_{i}y_{i},$$

where *w*_1_, *w*_2_, ···, *w*_*n*_ are weights that need to be found. The question boils down to how to weight nearby samples of the prediction point in question, and this is pertinent to other whole-genome prediction methods as well. Predicted values are represented as a weighted sum of the observed phenotypes and are functions of a projection matrix **H**. For example, BLUP and kernel ridge regression are represented as **ŷ**_*BLUP*_ = **H**_*BLUP*_**y** and **ŷ**_*RKHS*_ = **H**_*RKHS*_**y**, respectively, where **H**_*BLUP*_ = [**I** + **G**^−1^ λ]^−1^ and **H**_*RKHS*_ = **K**[**K** + λ**I**]^−1^. Weights may change as we move along when predicting a response at next unobserved vertex in kriging.

In the context of prediction of a random variable, an unbiased predictor requires that the expected value of the difference between predictor and predictand be zero. If the predictor is linear as in Equation (12),

(13) $$\begin{array}{l} E[{\hat{y}}_{0}-y_{0}]=E\left[\sum_{i\;=\;1}^{n}w_{i}y_{i}-y_{0}\right]\\ \;=\sum_{i\;=\;1}^{n}w_{i}E[y_{i}]-E[y_{0}]\\ \;=\mu \sum_{i\;=\;1}^{n}w_{i}-\mu , \end{array}$$

where (assuming no nuisance parameters) μ is the mean values of the responses; *E*[*y*_*i*_] = *E*[*y*_0_] = μ holds because of the stationarity assumption made. Setting Equation (13) to zero gives the unbiasedness condition

(14) $$\mu_{1}\left(\sum_{i\;=\;1}^{n}w_{i}-1\right)=0.$$

Thus, *ŷ*_0_ is an unbiased predictor if $\sum_{i=1}^{n}w_{i}=1$. This is called the “normed weights” condition.

As defined by Henderson (1973), BLUP is a linear unbiased predictor with minimum prediction error variance in such class. Under the unbiasedness condition, this is attained by minimizing the expected squared prediction error.

(15) $$\begin{array}{l} Var\left(\left[{\hat{y}}_{0}-y_{0}\right]\right)=E{\left[{\hat{y}}_{0}-y_{0}\right]}^{2}\\ \;=Var\left[\sum_{i\;=\;1}^{n}w_{i}y_{i}-y_{0}\right] \end{array}$$

Adding the unbiasedness conditions to the above equation via a Lagrange multiplier (λ) and setting the *n* + 1 partial first derivatives with respect **w** and λ to 0, one obtains the kriging system of linear equations shown below.

(16) $$\underset{\left(n\;+\;1)\times (n\;+\;1\right)}{\underset{︸}{\left[\begin{array}{cc} V+I\sigma_{\epsilon }^{2} & 1^{\prime }\\ 1 & 0 \end{array}\right]}}\cdot \underset{(n\;+\;1)\times 1}{\underset{︸}{\left[\begin{array}{c} \hat{\text{w}}\\ \lambda \end{array}\right]}}=\underset{(n\;+\;1)\times \;1}{\underset{︸}{\left[\begin{array}{c} C_{0i}+I\sigma_{\epsilon }^{2}\\ 1 \end{array}\right]}},$$

where the left-hand side includes the covariance function **V** = {**Cov**(**g**(**x**_**i**_), **g**(**x**_**j**_))}, the residual covariance matrix **I**σ^2^_*e*_, the vector of ones **1**. The right-hand side contains covariances between the unsampled location to be predicted and sampled locations **C**_0**i**_ = {**Cov**(*g*(*x*_0_), **g**(**x**_*i*_))}. We predict a value at some unsampled vertices by replacing the unknown weights with **ŵ**. Although derived from a different perspective, the predicted value *ŷ*(*x*_0_) is essentially BLUP of Henderson (Robinson, 1991): namely, the projection of yet-to-be-observed data on a linear combination of observed data. Ober et al. (2011) applied two alternatives to this ordinary kriging, named simple kriging and universal kriging, in the context of whole-genome prediction. Kriging has also been used for predicting human disease outcomes by condensing genomic and RNA expression data into kernel matrices (Wheeler et al., 2014). It is worthwhile to note that kriging is equivalent to Gaussian process regression in the machine learning literature (Rasmussen and Williams, 2005). In summary, BLUP in animal breeding, RKHS in statistics, kriging in geostatistics, and Gaussian process regression in machine learning all share the same spirit.

## 3. Kernel matrices

A suite of kernel functions has been proposed for whole-genome prediction purposes. Here, we discuss several kernels that can be used conjunction with the aforementioned regression methodologies. All kernels are a special case of a matrix denoted as **K**. A “parametric” kernel aims to capture the signal from some specific gene action, e.g., dominance. This approach permits making claims or interpretation with respect to some theory about hidden genetic architecture. At the other end of the spectrum, use of a “non-parametric” kernel is purely driven by prediction purposes. The kernel may pick up genetic signals regardless of the underlying genetic architecture, and model coefficients typically do not have a theoretical interpretation.

### 3.1. Parametric kernels

Most of the relationship matrices animal breeders have been using for many years are valid RKHS kernels, e.g., the additive genetic relationship matrix **A**, which is calculated directly from pedigree information. The idea here is to use expectation of genetic relatedness in the absence of genomic information as kernel. The off-diagonals of this matrix are twice the kinship coefficients, and 1 + individual's inbreeding coefficients are placed along the diagonal (Wright, 1922; Malécot, 1948). This is the perhaps the oldest kernel function used in quantitative genetics and is proportional to identical by descent (IBD) probabilities. Similarly, one can also trace expected dominance relationship coefficients using a pedigree (Henderson, 1985). A quantitative trait loci (QTL)-based counterpart of the additive genetic relationship matrix gained attention afterwards (Fernando and Grossman, 1989; Nejati-Javaremi et al., 1997; Villanueva et al., 2005).

The genomic relationship matrix **G** used in GBLUP also represent parametric kernels, using additively coded genotypes. Other types of genomic relationship matrices have been compared and discussed by Toro et al. (2011). As stated earlier, molecular similarity generates covariance even if individuals are not related in the sense of pedigree. Contrary to pre-genomics quantitative genetics, built largely on related individuals, the use of genomic data without genetic relatedness expands quantitative genetics theory (e.g., Yang et al., 2010). Analogous to **G**, the dominance counterpart **D** is constructed by setting up an appropriate dominance contrast between genotypes, for example, AA = −1, Aa = 0, and aa = −1 (Su et al., 2012; Vitezica et al., 2013; Da et al., 2014).

### 3.2. Non-parametric kernels

The genomic relationship matrix of VanRaden (2008) represents identical by state (IBS) similarities so there is no requirement to trace back genealogy. It is possible to compute an IBS matrix non-parametrically to enhance predictive performance. It is desirable to pick a kernel matrix that captures characteristics of the data. Such kernels allow interpreting classical relatedness as genomic spatial distances, and are expected to capture some of the complexity of the genome, including non-additive effects. Smoothing of the relatedness encoded by **G** may yield better predictions under complex gene action. We cover a wide variety of kernels that are non-linear in SNPs genotype codes, but appear as linear in a regression model as seen in Equation (6).

In a Gaussian kernel, we embed individuals in the Euclidean space, and the corresponding metric is a squared Euclidean norm. For example, the spatial genetic distance between individuals (*i, j*) is given by

$$\begin{array}{l} K(x_{i},x_{j})=\exp\left(-\theta d_{ij}^{2}\right)\\ \;=\prod_{k\;=\;1}^{m}\exp\left(-\theta {\left(x_{ik}-x_{jk}\right)}^{2}\right) \end{array}$$

where θ is a positive bandwidth parameter, $d_{ij}=\sqrt{{\left(x_{i1}-x_{j1}\right)}^{2}+\cdots +{\left(x_{ik}-x_{jk}\right)}^{2}+\cdots +{\left(x_{im}-x_{jm}\right)}^{2}}$ is the Euclidean distance, and *x*_*ik*_ (*i, j* = 1, ···, *n, k* = 1, ···, *m*) is the SNP genotype code for individual *i* at SNP *k*. The smaller the Euclidean distance is, the stronger the similarity in state between two genotype vectors. Taking exponentiation of the negative Euclidean distance changes the direction of relatedness, that is, a larger distance produces a smaller value of the Gaussian kernel and a smaller spatial genetic similarity. Gianola et al. (2006) introduced the Gaussian kernel in quantitative genetics with the aim of capturing total genetic effects in a whole-genome prediction problem. This kernel is known to be a particular case of what is called a Gaussian radial basis function (RBF). The Gaussian RBF computes spatial genetic distance between *n* individuals and *n*′ individuals chosen as centroids (*n* > *n*′). Some applications of this in the context of genome-enabled prediction are in Long et al. (2010) and González-Camacho et al. (2012). While the idea is to choose a minimal set of basis functions, the resulting kernel is no longer semi-positive definite from the RKHS point of view. This approximation approach may help greatly when *n* is large. When *n* is chosen to be equal to *n*′, this leads to a standard Gaussian kernel.

The exponential kernel first explored in Piepho (2009) is closely related to the Gaussian kernel. It takes the form exp(−θ*d*_*ij*_), so the squared norm is dropped. The Matérn covariance function is a generalization of an Euclidean distance-based kernel that contains the Gaussian and the exponential kernels as particular cases. An advantage of this function is that the actual form of a kernel can be inferred from the data directly, permitting flexible kernel selection. Based on simulated data, the Gaussian kernel appeared to give the best fit within this specific class of kernel (Ober et al., 2011).

Given that SNPs take discrete values only, it seems reasonable to remove redundant areas in the Euclidean space that are never used. The diffusion kernel, which is a discretized Gaussian kernel, can be viewed as functions on discrete spaces, such as a graph. Morota et al. (2013) employed the SNP grid kernel, that is, the diffusion kernel specifically developed to model SNP data distributed on a grid graph, as described earlier. This measures how similar two vertices are in terms of Manhattan distance on an *m*-dimensional grid graph. The essence of this kernel is the matrix exponentiation of a graph Laplacian. The SNP grid kernel between two vertices consisting of spatial SNP coordinates on the *p*-dimensional grid graph is given by

(17) $$\begin{array}{l} K_{\theta }(x,x^{\prime })\propto {\left(\frac{-2e^{-3\theta }+2}{e^{-3\theta }+3e^{-\theta }+2}\right)}^{n_{1}}{\left(\frac{e^{-3\theta }-3e^{-\theta }+2}{e^{-3\theta }+3e^{-\theta }+2}\right)}^{n_{2}}\\ \;{\left(\frac{4e^{-3\theta }+2}{e^{-3\theta }+3e^{-\theta }+2}\right)}^{m_{11}} \end{array}$$

where *n*_*s*_ is the number of SNPs at which the copy of the “A” allele between two individuals differ by *s*, and *m*_11_ is the number of SNPs at which two individuals share heterozygous states. The bandwidth parameter θ > 0 controls a rate of diffusion (degree of relatedness). A diffusion kernel for binary genotypes (presence or absence) is likewise constructed as

(18) $$K_{\theta }(x,x^{\prime })\propto {\left(\frac{1-\exp(-2\theta )}{1+\exp(-2\theta )}\right)}^{d(x,x^{\prime })}$$

where *d*(**x**, **x**′) is the Hamming distance, that is, number of coordinates at which **x** and **x**′ differ. Morota et al. (2013) evaluated the performance of the diffusion and of the Gaussian kernels using dairy cattle and wheat line data. It turned out that differences in predictive ability were negligible, suggesting that the simple Gaussian kernel is very robust.

A similar attempt at refining the Gaussian kernel is in Tusell et al. (2014). These authors proposed a *t* kernel for *m* markers taking the form

$$K(x_{i},x_{j})\;={\left[1+\frac{\left(x_{i}-x_{j})\prime \Sigma^{-1}(x_{i}-x_{j}\right)}{m\nu }\right]}^{-\frac{\left(\nu \;+\;m\right)}{2}},$$

where Σ is an *m* × *m* scale matrix and ν is the degrees of freedom (a positive continuous parameter acting as bandwidth). The authors used a diagonal matrix for Σ^−1^, with its *k*th element equal to the heterozygosity at the *k*th SNP locus: 2*p*_*k*_ (1 − *p*_*k*_), and evaluated the kernel performance over a fixed grid of values of ν. This kernel aims to expand the underlying metric space with its heavier tails. The *t* kernel resulted in a similar performance as the Gaussian kernel (Tusell et al., 2014) suggesting that the avenues for enhancing predictive ability through kernel refinement are limited, in agreement with Morota et al. (2013). The picture that emerges here is that use of the Gaussian kernel is probably sufficient for a prediction task.

Covariance functions over the prediction grid need to be specified for interpolation when applying kriging and Gaussian process regression. In this setting, the kernel matrix **K** is the covariance matrix of a stochastic process. Here, in order to explicitly interpret a kernel as a covariance function, we assume E(*g*(*x*_.._)) = 0. Then the covariance function becomes *Cov*(*g*(*x*_.._), *g*(*x*′_.._)) = E(*g*(*x*_.._), *g*(*x*′_.._)) = *K*(*g*(*x*_.._), *g*(*x*′_.._)), namely, a kernel.

It is worth keeping in mind that the kernel defines the the inner product, the inner product defines the covariance, and the covariance brings up a new metric called Hilbert space (distance). While some simply structured kernels such as the IBS kernel (Wessel and Schork, 2006), the weighted IBS kernel (Kwee et al., 2008; Wu et al., 2010), and the Wright-Fisher kernel (Zhu et al., 2012) have been applied for GWAS purposes in human genetic epidemiology contexts, their use in animal and plant quantitative genetics has been very limited. In general, constructing non-parametric kernel matrices is computationally more taxing than for their parametric counterparts.

### 3.3. Kernel averaging

Kernel methods do not preclude use of several kernels together. An alternative approach is to use “kernel averaging” or “multiple kernel learning,” as proposed in de los Campos et al. (2010). Suppose there are three kernels **K**_1_, **K**_2_, and **K**_3_ that are distinct from each other. In this approach, the three kernels are “averaged” to form a new kernel $K=K_{1}\frac{\sigma_{K_{1}}^{2}}{{\tilde{\sigma }}_{\text{K}}^{2}}+K_{2}\frac{\sigma_{K_{2}}^{2}}{{\tilde{\sigma }}_{\text{K}}^{2}}+K_{3}\frac{\sigma_{K_{3}}^{2}}{{\tilde{\sigma }}_{\text{K}}^{2}}$, where σ^2^_*K*_1__, σ^2^_*K*_2__, σ^2^_*K*_3__ are variance components attached to kernels **K**_1_, **K**_2_, and **K**_3_, respectively, and ${\tilde{\sigma }}_{\text{K}}^{2}$ is the sum of the three variances. The ratios of the three variance components are tantamount to the relative contributions of the kernels to the marked genetic variation in the population. For instance, the kernels used can be three Gaussian kernels with different bandwidth parameter values, as employed in Tusell et al. (2014), or one can fit several parametric kernels jointly, e.g., the additive (**G**), dominance (**D**), and additive by dominance (**G#D**) kernels as in Morota et al. (2014b). While there are many possible choices for kernels, the kernel function can be estimated via maximum likelihood by recourse to the Matérn family of covariance function (e.g., Ober et al., 2011) or by fitting several candidate kernels simultaneously through multiple kernel learning.

## 4. Applications of kernel methods

A long standing question in quantitative genetics is how important epistasis is for complex traits. To a large extent, animal breeding focuses on additive variability. On the other hand, there is increasing evidence that complex traits are the product of synergistic forces spanned by a large number of genetic polymorphisms along the genome and, theoretically, functional epistasis can play an important role in selection response (Hansen, 2013). Kernel methods are theoretically appealing for accommodating cryptic forms of gene action (Gianola et al., 2006; Gianola and van Kaam, 2008).

### 4.1. Whole-genome prediction

Kernel-based whole-genome prediction is being increasingly employed across many species. Within this class of methods, statistical models are linear in the parameters but may be non-linear in the covariates. We gather some of case studies in the published literature here, although not in great depth. The theoretical framework of RKHS regression for genome-enabled prediction first appeared in Gianola et al. (2006), and was subsequently more firmly characterized in Gianola and van Kaam (2008). González-Recio et al. (2008, 2009) reported early applications of the procedures to data on mortality and food conversion rate in broiler chickens. These authors compared RKHS regression with various linear additive smoothers and concluded that semi-parametric methods had potential for capturing total genetic effects from real data. While same superiority of semi-parametric kernel methods over additive models has been found for body weight of broiler chickens (Long et al., 2010), differences were minimal when applied to litter size in swine (Tusell et al., 2013), dairy sires progeny test (Long et al., 2011; Morota et al., 2013), and phenotypes of dairy cows (Morota et al., 2014b). While various predictive models have not differed substantially, on average, some of the semi-parametric approaches, including RKHS, have performed consistently better.

Applications of semi-parametric regressions in plant breeding have also produced encouraging results. The semi-parametric approach was evaluated in plant breeding by Crossa et al. (2010) who compared RKHS with Bayesian LASSO using CIMMYT data sets. Therein, 599 wheat lines genotypes with 1447 markers and 300 tropical maize lines genotyped with 1148 SNPs were analyzed for this purpose. While RKHS and Bayesian LASSO showed a similar predictive ability in maize, the former outperformed the latter in the wheat lines. This wheat data set has been used in many subsequent studies, and the apparent advantage of RKHS regression was confirmed (de los Campos et al., 2010; Endelman, 2011; Long et al., 2011; Heslot et al., 2012; Morota et al., 2013; Tusell et al., 2014). Heslot et al. (2012) performed an extensive comparison of prediction methods including RKHS, neural networks, support vector machines, ridge-regression BLUP, and random forests. RKHS topped 15 out of 18 trait-comparisons and performed the best on average in terms of predictive correlations. González-Camacho et al. (2012) compared RKHS, radial basis function neural networks (RBFNN) and the Bayesian LASSO using 21 trait-environment combinations of maize data sets. Although the differences were small, RKHS had a consistently higher predictive correlation than RBFNN and the Bayesian LASSO. Subsets of these maize data were reanalyzed in another exhaustive comparison carried out by Ornella et al. (2014). Among six regression methods, RKHS ranked as the best prediction machine when applied to 14 maize data sets and across trait-environment combinations as measured by Pearson's correlation coefficient. Echoing work of González-Camacho et al. (2012), Pérez-Rodríguez et al. (2012) tested RKHS, RBFNN, Bayesian regularized neural networks, and linear additive smoothers, including Bayesian LASSO, Bayesian ridge regression, BayesA, and BayesB. BayesA and BayesB are marker-based Bayesian hierarchical linear regression models developed to capture additive genetic effects of markers (Meuwissen et al., 2001). The data used were 306 elite wheat lines coupled with 1717 diversity array technology (DArT) markers. The authors observed a consistent superiority of non-additive smoothers over additive counterparts, yet, RKHS and RBFNN were equally competitive. Kernel-based whole-genome prediction models have also been applied to genotyping-by-sequencing on maize populations (Crossa et al., 2013). Overall, RKHS performed slightly better than GBLUP when genomic information was the sole source of information used for predictors. A study led by Sun et al. (2012) reported that RKHS and smoothing spline ANOVA model (alternative parametrization of Bayesian kernel ridge regression) coupled with supervised principal component analysis delivered slightly higher predictive correlations in barley and maize data. On the other hand, no clear difference was observed between RR-BLUP and RKHS, which led the authors to conjecture that there appear to be no epistatic effects on six maize traits studied (Riedelsheimer et al., 2012b). In retrospect, RKHS is at least as good as linear additive smoothers: on one hand it delivers better predictive performance when non-additive effects are present and, on the other hand, produces a similar performance when additivity is the main source of genetic variation.

### 4.2. Assessment of predictive performance

Avoiding overfitting of prediction models is desirable because one wishes to extract genetic signal but not noise. Since its introduction into animal breeding by Meuwissen et al. (2001), cross-validation (CV) has quickly become the technique of choice for measuring prediction performance. It is being widely used in whole-genome prediction and there are a number of ways of applying it. For instance, CV designs include: (1) two-generation (stratification by generation or date of birth) validation, (2) k-fold validation, and (3) repeated random sub-sampling validation. We highlight here pros and cons of each. The two-generation scheme is a reasonable choice to use when a data set comprises parents and their offspring. The records on parents are used to train the model and prediction is carried out in a testing set that includes offspring of individuals in the training set. While this setting can simulate a standard genetic evaluation scenario and prevent an unrealistic case such as predicting parent responses from offspring records, it generates only a single realized prediction accuracy measurement, and an alternative is to bootstrap the layout. In k-fold validation scheme, the entire data set is first randomly splitted into k disjoint subsets of equal size. Within each fold, k-1 subsets are used to fit the model and the remaining subset is used as a testing set to predict masked phenotypes of individuals. This is repeated until all k subsets are used as testing and results from the k-fold are averaged. Typically, k = 5 or 10 is used to assess predictive ability. Unlike two-generation validation, an advantage of this approach is that it is possible to estimate CV uncertainty but at the cost of higher computation load. Recent research (Makowsky et al., 2011; Saatchi et al., 2011; Pérez-Cabal et al., 2012; Kramer et al., 2014) have shown that the k-fold CV gives a slightly better predictive performance than a two-generation validation. In practice, the design of CV must keep the target problem in mind. Lastly, repeated random sub-sampling validation randomly partitions the data set into training (e.g., 90%) and testings (e.g., 10%) sets. Then, an average of say, 50 random repeats of the cross-validation is computed, and this CV layout has been adopted in some past studies (e.g., González-Camacho et al., 2012; Pérez-Rodríguez et al., 2012; Gianola et al., 2014b). While it also permits to obtain a CV distribution, some individuals may never enter into training and testing sets or appear more often than others. While these three CV strategies are widely adopted in practice, it is important to note that the ultimate goal is to successfully predict in cross-study validation using independent data.

Prediction performance on CV in the testing set can be assessed by Pearson's correlation or via the predictive mean-squared error between observed and predicted values. A squared predictive correlation aims to measure the amount of variation captured by prediction. However, prediction assessment is not limited to these two measures; for example, Ornella et al. (2014) used Cohen's kappa coefficient (Cohen, 1960) and found that it provided similar indicators of the performance of genome-enabled prediction when selecting the best individuals. On the other hand, the behavior of prediction machines differed when the aim was to separate the best and worst individuals, e.g., selecting the best 15% of individuals. This suggests that optimal prediction methods that perform well when predicting individuals in the testing set overall, may result in a poor performance when the target is only the tails of the distribution in the same testing set (Ornella et al., 2014).

Assessment of predictive performance is not exclusively limited to use of CV. Stone (1977) showed that leave-one-out CV and Akaike's information criterion (AIC) are asymptotically equivalent when maximum likelihood estimation is used. An underappreciated point is that Akaike developed AIC to quantify goodness-of-prediction rather than goodness-of-fit (Akaike, 1974). By leveraging this property, it is possible to evaluate predictive ability of models without conducting computationally expensive CV. The prediction model associated with the smallest AIC is considered to fit the data well from a predictive point of view. Note that the proof in Stone (1977) is not restricted to linear models. Piepho (2009) assessed predictive ability of several models using AIC, and the study carried out by Schulz-Streeck and Piepho (2010) found congruence between AIC and the predictive correlation (the smaller the AIC values, the higher the predictive correlations). That said, this pattern did not always hold, suggesting that the CV remains as a viable tool to gauge predictive performance.

Another avenue is to derive deterministic equations that compute expected predictive correlation on the basis of a number of factors. These include size of the training data set, the effective number of independent chromosome segments, the number of markers used, and heritability of the trait (Daetwyler et al., 2008, 2010; Goddard, 2009; Goddard et al., 2011; Erbe et al., 2013). In relation to these, de los Campos et al. (2013b) gave a theoretical upper limit for the achievable predictive ability that does not use number of independent chromosome segments. This is appealing because the number of independently segregating segments is a somewhat idealized concept and not straightforward to estimate. It may be argued that it is worthwhile to employ AIC or deterministic equations prior to performing resource intensive CV, to see what can be expected empirically.

### 4.3. GWAS-based prediction

The first large-scale genome-wide association study (GWAS) involving hundreds of thousands of SNPs was reported by Ozaki et al. (2002). The authors identified some functional variants associated with myocardial infarction using 92,788 genotyped SNPs in more than 2000 individuals. Since then, a number of GWAS results have been reported (Visscher et al., 2012), and the success of GWAS seems largely due to the biochemical technology that generates high dimensional markers spanning the entire genome, rather than the statistical methodologies that have been employed.

Genome-enabled prediction of traits outside of animal and plant domains has been mainly carried out by targeting specific genes or variants identified from inference procedures (GWAS or their meta-analyses) as opposed to using all available markers. This indicates that deciding which genetic variants to include in the prediction equation is a crucial component. In this line, Morota et al. (2014a) classified SNPs based on functional annotation and created kernels for each of several genomic regions. They observed that functionally annotated regions did not always deliver a better predictive performance than intergenic regions in three broiler traits analyzed. A whole-genome regression model incorporating all available quality filtered SNPs attained a similar performance to that from the genomic region (either genic or intergenic) that achieved the best prediction. Likewise, results in Holstein cattle obtained by Erbe et al. (2012) found that the predictive performance of the whole ensemble of SNPs was comparable to that of markers in exonic regions. While the whole-genome prediction approach appears to be adequate for practical purposes, to what extent preselection of SNPs residing in functionally enriched regions aids predictive ability is a subject for further investigation. One plausible explanation for the observed high predictive performance of intergenic regions is in Schierding et al. (2014). Recall that all chromosomes are folded such that certain genic regions (e.g., exons) may physically interact with distantly located gene deserts in the sense of a 3-dimensional space. This creates spatial associations and may allow intergenic regions to regulate gene function from far away on a linear scale.

Most GWAS rely on *p*-values derived from a series of single marker regressions or with inclusion of a genomic relationship matrix to correct for false positive associations due to population structure or relatedness. A new approach, kernel-based GWAS, appears to be emerging. For example, Maity et al. (2012) reported that a multivariate kernel GWAS can potentially accommodate interaction and non-linear effects among markers. However, a pitfall of the two-steps approach (preselection of predictors) based on *p*-values has been studied by Lazzeroni et al. (2014), leading to the conclusion “While uncertainty is high for a *p*-value from a single test, *p*-values obtained from GWAS, or other multiplexed studies requiring multiple testing corrections, provide almost no information with which to make future predictions.” due to the high variability of *p*-values. This suggests that if associated variants are simply picked by non-replicable *p*-values in GWAS, the resulting prediction step will fail (Wray et al., 2007). Currently, GWAS in animals is predominantly carried out as a by-product of genome-enabled prediction, in which the degree of association is estimated from marker effect sizes. While an ultimate goal is to dissect genetic architecture and to make predictions from validated genetic variants, whether knowledge from “inference” can aid prediction is yet to be answered. Arguably, the future may reside on inference, but there is plenty of room for connecting inference and prediction. Additional criteria need to supplement the use of *p*-values (Malley et al., 2013). Perhaps, there is a critical need to adopt more CV in GWAS. Significance of detected variants in one data set should be reaffirmed in a separate data set. This is because the fraction of genetic variance explained by QTL or by a subset of markers in a testing set is much less than that of the training set (e.g., Utz et al., 2000; Makowsky et al., 2011; Würschum and Kraft, 2014).

## 5. Conclusions

We reviewed the utility of kernel methods as a tool of choice for a prediction of yet-to-be observed phenotypes, and described their relation to spatial variation as determined by a vector of SNPs. Research aiming to extend Fisher's infinitesimal model to accommodate non-additive effects either parametrically or non-parametrically is a topic of interest. Most studies carried out so far suggest that whole-genome prediction coupled with combinations of kernels may capture non-additive variation (Gianola et al., 2014a; Howard et al., 2014). These approaches are also applicable to whole-genome risk prediction by introducing the concept of liability (Wright, 1934; Falconer, 1965; Gianola, 1982). In many applications, accurate prediction of individual responses rather than of breeding values is a primal interest.

Many challenges remain, however. We conclude by highlighting some potential future directions in kernel-based whole genome prediction methods. Throughput of genomics, transcriptomic and proteomic technologies has advanced tremendously in recent years. Arguably, emerging molecular information makes quantitative genetics more powerful because it permits to expand a theory that was initially largely built around pedigree information during the last century. It could be argued that 21st century quantitative genetics needs to be linked to functional genomics, and not simply relying on hypothetical QTL that may or may not exist.

In addition, more study on methodology specifically tailored for emerging omics data that combines statistical approaches and functional validation is required. For example, the RKHS method accommodates any information set for input variables. This paves the way to link phenotype and genome jointly with intermediate phenotypes such as transcriptomic, proteomic and metabolomic data in a single framework, known as systems genetics (e.g., Civelek and Lusis, 2013). Recent attempts along this line pertinent to genome-enabled prediction include joint evaluations of genome and transcriptome (Bhattacharjee and Sillanpää, 2011) and genome and metabolome (Riedelsheimer et al., 2012a). The question boils down to how we condense and construct kernels from each set of biological information. Also, estimation of non-additive variation in the eQTL context has been explored recently (Powell et al., 2013; Hemani et al., 2014).

Prediction of response variables is largely classified into predicting: (a) additive effects, (b) total genetic effects, and (c) raw phenotypes. The first type is mainstream in genome-enabled selection schemes, and we have given an overview of kernel methods in which their predominant aim is to capture total genetic effects. Although currently receiving less attention, predicting raw phenotypes is especially vital for assessing health or medical outcomes. Arguably, many non-genetic factors affect raw phenotypes, and these cannot be well predicted without environmental information, so imperfect information on environmental variables hinders achieving greater predictive performance (Heslot et al., 2014). One important direction for future study is that of condensing environmental variables into some “environmental kernel,” to cast a joint evaluation of genotype and environment via kernel methodology (Jarquín et al., 2014). Research involving analysis of raw phenotypes coupled with environmental variables needs more attention, and even when genetic selection is the sole interest, the use of pre-processed quasi-phenotypes (e.g., estimated breeding value) should be avoided, if possible, as reported by Ekine et al. (2014).

## Author contributions

Gota Morota and Daniel Gianola assembled relevant literature and wrote the manuscript.

### Conflict of interest statement

The authors declare that the research was conducted in the absence of any commercial or financial relationships that could be construed as a potential conflict of interest.
